# Using rivaroxoban in patients with venous thromboembolism

**DOI:** 10.1186/cc14402

**Published:** 2015-03-16

**Authors:** I Tyutrin, O Tarabrin, B Todurov, S Shcherbakov, D Gavrychenko, G Mazurenko

**Affiliations:** 1 Odessa National Medical University, Odessa, Ukraine

## Introduction

A prospective study was conducted in patients for treatment of venous thromboembolism (VTE) to compare the effect of enoxaparin and rivaroxaban using the method of low-frequency piezoelectric thromboelastography (LPTEG) for checking coagulation activation markers.

## Methods

A total of 60 patients entered the Odessa Clinical Regional Hospital for treatment venous thromboembolism. Patients were divided into two groups. The first group (*n *= 30) were receiving enoxaparin in dosage 1.5 mg/kg subcutaneously per day. The second group (*n *= 30) were receiving rivaroxaban orally 15 mg/day. For checking the coagulation state we were using such indicators of LPTEG as constant thrombin activity (CTA), intensity of coagulation drive (ICD) and gel point (GP). We performed LPTEG three times per day: 4, 12 and 24 hours after taking the drug to check for changes in the coagulation state in both groups of patients.

## Results

The peak action of enoxaparin and rivaroxaban was observed at 4 hours post administration. LPTEG indicators that determine the coagulation state after 4 hours in the first group: CTA was decreased by 72.12% (*P *< 0.05), ICD was decreased by 68.44% (*P *< 0.05), GP was increased by 17.9%; in the second group: CTA was decreased by 76.24% (*P *< 0.05), ICD was decreased by 74.52% (*P *< 0.05), GP was increased by 23.34%. After 12 hours, CTA in the first group decreased by 22.41%, ICD decreased by 5.3%, GP increased by 8.12%, indicating reduction of hypocoagulation effect; in the second group, CTA decreased by 39.35% (*P *< 0.05), ICD decreased by 40.24% (*P *< 0.05), GP increased by 18.25%. After 24 hours in the first group LPTEG indicators returned to the original value, and in the second group of patients CTA was decreased by 15.14%, ICD was decreased by 6.62%, GP increased by 14.22%.

## Conclusion

Using LPTEG showed the hypocoagulation effect of continuous rivaroxaban 24 hours after oral administration compared with enoxaparin, which retains less hypocoagulation effect 12 hours after administration. LPTEG indicators in the second group were bigger than in the first group after 12 hours: CTA 43.07%, ICD 69.72%, GP 54.12%.

